# Deletion of Snap25 disrupts glial remodeling in aging mouse brain

**DOI:** 10.1016/j.isci.2026.116478

**Published:** 2026-06-23

**Authors:** Auguste Vadisiute, Florina Szabo, Sofia Luchanskaya, Vanessa Drevenakova, Fernando Messore, Albert Ugwudike, Gretchen Greene, Marissa Mueller, Sophie V. Morse, Anna Hoerder-Suabedissen, Zoltán Molnár

**Affiliations:** 1 Department of Physiology, Anatomy and Genetics, Sherrington Building, University of Oxford, Oxford, UK; 2 St John’s College, University of Oxford, Oxford, UK; 3 Department of Bioengineering, Imperial College London, London, UK; 4 UK Dementia Research Institute, Imperial College London, London, UK; 5 Department of Computing, Imperial College London, London, UK; 6 Chelsea and Westminster Hospital NHS Foundation Trust, London, UK; 7 Kavli Institute for Nanoscience Discovery, Sleep and Circadian Neuroscience Institute, University of Oxford, Oxford, UK; 8 Sleep and Circadian Neuroscience Institute, University of Oxford, Oxford, UK; 9 National Institute of Neurological Disorders and Stroke, NIH, Bethesda, MD, USA

**Keywords:** neuroscience

## Abstract

Neuronal activity regulates glial physiology, but the effects of prolonged synaptic silencing in mature circuits are unclear. Using Rbp4-Cre-mediated Snap25 deletion to block neurotransmitter release in subsets of cortical layer 5 neurons and dentate gyrus granule cells, we examined glial responses across connected brain regions and the spinal cord in adult and middle-aged mice. Silenced cortical regions showed strong astrocytic reactivity and increased microglial density, while downstream targets, including the superior colliculus and CA3, exhibited marked microglial remodeling and synaptic changes. CA1 displayed milder alterations. In the spinal cord, microglial density decreased and GFAP^+^ astrocytes increased, whereas TNF-α levels and ChAT^+^ motor neurons were unchanged. Age amplified astrocyte heterogeneity and microglial reactivity under synaptic silencing. These findings suggest that circuit topology shapes the spatial and cellular specificity of glial responses to chronic loss of synaptic activity.

## Highlights

• Chronic synaptic silencing alters astrocyte and microglial states across brain
• Glial responses reflect circuit topology, with local and projection-specific effects
• Age amplifies glial morphological heterogeneity under reduced neuronal activity
• Spinal cord glial remodeling occurs without overt inflammation or motor neuron loss

## Graphical abstract

**Figure:**
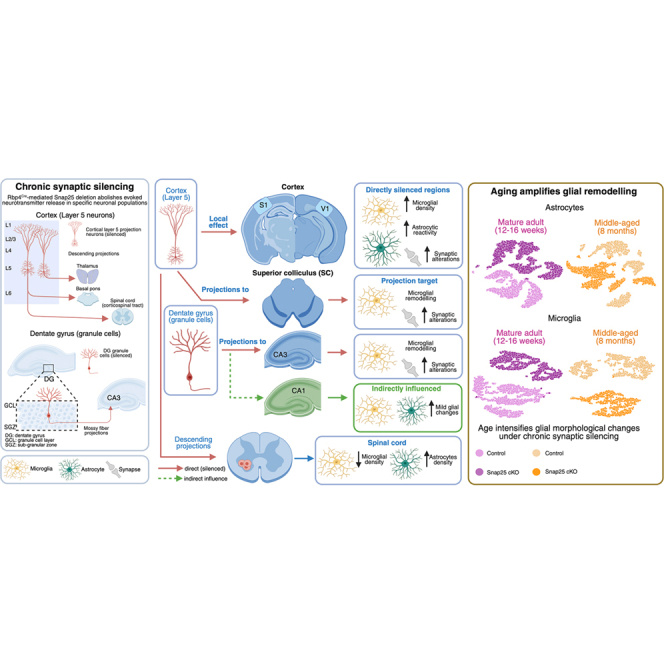

## Introduction

Neuronal networks form the fundamental information-processing architecture of the brain. However, their stability and function depend on continuous support from glial cells. Once considered passive elements, glia are now recognized as active regulators of synaptic signaling, metabolic homeostasis, and immune surveillance.^1^^,^^2^^,^^3^^,^^4^ Microglia and astrocytes play central roles in neurodevelopment, synaptic remodeling, and the maintenance of brain homeostasis.^5^ Dysfunction in either cell type contributes to neurodevelopmental disorders, including schizophrenia,^6^^,^^7^ as well as neurodegenerative conditions such as Alzheimer’s disease.^8^^,^^9^^,^^10^

While glial regulation of neuronal signaling is increasingly well established,^11^^,^^12^ the reciprocal influence of neuronal activity on glial states, particularly how defined patterns of synaptic output shape microglial and astrocytic behavior, remains incompletely understood. Both neuron-microglia and neuron–astrocyte interactions are fundamentally activity-dependent: neuronal firing directly regulates microglial process motility and surveillance^13^^,^^14^^,^^15^ and shapes astrocytic structural plasticity, metabolic coupling, and synaptic engagement.^16^^,^^17^^,^^18^^,^^19^ Targeted suppression of neuronal neurotransmission therefore provides a powerful approach to dissect neuron-glia communication *in vivo*.

Microglia, the resident immune cells of the brain, contribute to developmental circuit refinement through activity-dependent synaptic pruning^20^^,^^21^^,^^22^ and maintain adult brain homeostasis by dynamically surveying their microenvironment in a neuronally regulated manner.^13^^,^^23^^,^^24^^,^^25^^,^^26^ Astrocytes likewise exhibit pronounced activity-dependent plasticity. As core components of tripartite synapses,^27^ astrocytes adapt their synaptic coverage in response to neuronal activity,^16^ migrate following loss of neuronal function, regulate neurotransmitter and pH homeostasis in an activity-sensitive manner,^16^^,^^28^^,^^29^^,^^30^^,^^31^ and engulf synapses in an activity-dependent fashion.^17^^,^^32^ Astrocyte reactive states involve coordinated morphological, transcriptional, and functional changes,^33^^,^^34^ with markers such as GFAP and S100B frequently—but not uniformly—associated with astrocyte reactivity.^35^^,^^36^

Despite extensive evidence that glial cells respond to neuronal activity, the mechanisms by which sustained alterations in synaptic output shape glial phenotypes across defined circuits remain poorly resolved. To address this gap, we employed a genetic strategy to selectively disrupt activity-dependent neurotransmission and examine the resulting glial responses across cortical, hippocampal, and spinal circuits.

SNAP25 (synaptosomal-associated protein 25) is a core component of the SNARE complex^37^^,^^38^^,^^39^ and is essential for calcium-evoked synaptic vesicle fusion, and global deletion results in perinatal lethality,^37^ demonstrating the necessity of evoked neurotransmission for survival. Although spontaneous neurotransmitter release persists in the absence of SNAP25,^40^ evoked synaptic transmission is effectively abolished. Conditional deletion of *Snap25* enables chronic suppression of regulated vesicular release in genetically defined neuronal populations while preserving early structural development.^37^^,^^41^^,^^42^

In the present study, we used an Rbp4-Cre-driven *Snap25* conditional knockout (cKO) targeting corticofugal layer 5 pyramidal neurons and dentate gyrus granule cells.^43^ In this model, Cre expression begins embryonically and drives *Snap25* deletion from early postnatal stages onward. Axonal degeneration becomes apparent around postnatal day 21, followed by progressive terminal loss and quantifiable neuronal death by approximately 8 months of age.^43^ This model therefore allows investigation of both local effects of synaptic silencing and projection-specific consequences in downstream regions such as the superior colliculus and hippocampal CA fields, while CA1—lacking direct input from the silenced populations—serves as an indirect, network-influenced region.

Based on the role of neuronal activity in regulating glial physiology, we hypothesized that chronic loss of evoked synaptic transmission via Snap25 deletion would induce circuit-specific glial remodeling at the site of the cell bodies and at the sites of the termination of their projections. Specifically, we predicted that regions that contained the neurons that were directly subjected to synaptic silencing would exhibit the most pronounced astrocytic and microglial changes, whereas downstream projection targets would display distinct secondary adaptations, and indirectly connected regions would show more modest alterations. Furthermore, given the progressive circuit disruption described in this model,^43^^,^^44^ we hypothesized that these effects would be exacerbated with age, reflecting reduced homeostatic resilience in the aging brain.

By leveraging loss of Snap25 to block evoked synaptic vesicle release, we demonstrate that activity-dependent neurotransmission is essential for maintaining glial organization, morphology, and reactivity state. Our findings reveal that glial responses to sustained synaptic silencing are shaped by circuit topology, age, and cellular context, highlighting the importance of neuronal activity in preserving glial homeostasis across the brain and spinal cord.

## Results

**Figure 1:**
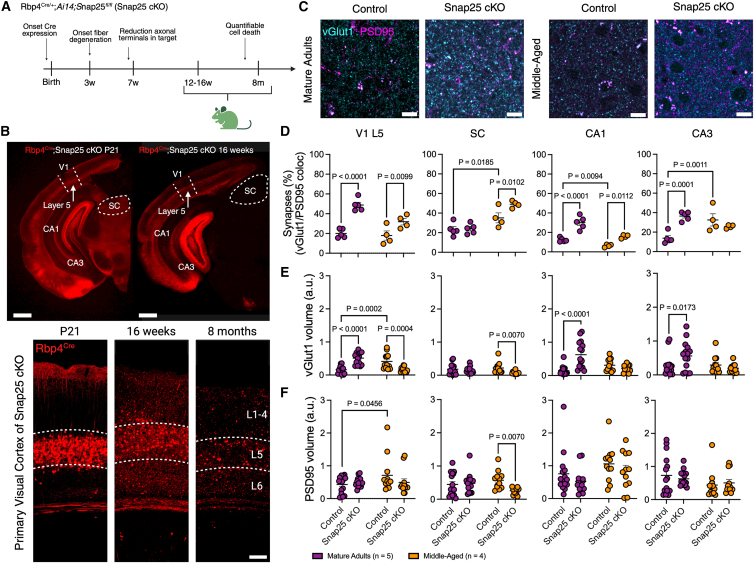
Loss of Snap25 alters synaptic density in mature and middle-aged mouse brains (A) Summary of the Snap25 cKO mouse line showing the onset of fiber degeneration and the timeline of neurodegenerative events leading to cell death in the Snap25 cKO strain. Animals aged 12–16 weeks and 8 months were used in this study. (B) Representative images of Rbp4Cre-silenced neurons (red) at different ages illustrate progressive cell death. (C) Representative images of synaptic densities in mature adult and middle-aged mouse brains. Presynaptic compartments were labeled with vGlut1 (cyan), and postsynaptic compartments were labeled with PSD95 (magenta). (D–F) Quantification of synaptic density and presynaptic compartment size in mature adult (purple) and middle-aged (orange) control vs. Snap25 cKO mice. Mice used: mature adults, *n* = 5 (control) and *n* = 5 (Snap25 cKO); middle aged, *n* = 4 (control) and *n* = 4 (Snap25 cKO). Synaptic density and vGlut1 and PSD95 puncta size were measured in layer 5 of the primary visual cortex. Data were analyzed using mixed-effects ANOVA with Fisher’s LSD test. All data are presented as mean ± SEM. Statistical significance is indicated in the images. Scale bars (B and C): 100 μm.

We analyzed Rbp4-Cre;Snap25fl/fl;Ai14 (Snap25 cKO) mice at 12–16 weeks (young/mature adults) and at 8 months (middle aged). Progressive axonal degeneration and subsequent neuronal loss in this model have been described previously.^43^ In line with this established timeline, we observed degeneration of axonal projections over time, followed by a reduction in labeled neuronal profiles at later stages (Figures 1A and 1B). At postnatal day 21 (P21), tdTomato-labeled Rbp4-Cre-expressing neurons were robustly detected in cortical layer 5. By 8 months of age, the tdTomato signal appeared more diffuse across cortical layers, consistent with fragmentation and disintegration of axonal terminals and the accumulation of labeled debris rather than an expansion in the number of labeled neurons (Figure 1B).

### Loss of Snap25 alters synaptic density and size in an age- and region-dependent manner

To determine how abolishing Snap25-dependent evoked neurotransmitter release affects synaptic organization, we visualized excitatory synapses using 3D confocal microscopy and quantified synaptic puncta as the co-localization of presynaptic vGlut1 and postsynaptic PSD95. Analyses were performed in layer 5 of the primary visual cortex (V1 L5), the superior colliculus (SC), and the CA1 and CA3 regions of the hippocampus in mature adult (12–16 weeks) and middle-aged (8 months) mice.

We first assessed age-related changes in synaptic density. Age-related effect was observed in V1 L5 (F(1,14) = 9.043, *p* = 0.0094), SC (F(1,14) = 34.44, *p* < 0.0001), CA1 (F(1,6) = 89,16, *p* < 0.0001) but not in CA3 (F(1,6) = 3.320, *p* = 0.1183, Figure 1D). In control mice, synaptic density increased with age in SC (*p* = 0.0185) and CA3 (*p* = 0.0011) but decreased with age in CA1 (*p* = 0.0094) while V1 L5 showed no significant age-dependent differences (Figure 1D).

We next examined the effects of Snap25 deletion. In V1 L5, synaptic density was significantly increased in Snap25 cKO mice compared with controls (genotype effect: F(1,14) = 45.77, *p* < 0.0001, Figure 1D), with elevated density observed in both young/mature adults (*p* < 0.0001) and middle-aged animals (*p* = 0.0099). In the SC, synaptic density was also increased in Snap25 cKO mice (F(1,14) = 5.387, *p* = 0.0359, Figure 1D), but this effect was restricted to middle-aged animals (*p* = 0.0102). In CA1, synaptic density was elevated in Snap25 cKO mice (F(1,8) = 27.93, *p* = 0.0007, Figure 1D) and this effect was observed in mature adults (*p* < 0.0001) and middle aged (*p* = 0.0112). In CA3, genotype effect was not statistically significant (F(1,8) = 4.429, *p* = 0.0684, Figure 1D) but synaptic density was increased only in mature adults (*p* = 0.0001). Furthermore, age and genotype effect was observed in V1 L5 (F(1,14) = 5.391, *p* = 0.0358), CA1 (F(1,6) = 16.97, *p* = 0.0062), and CA3 (F(1,6) = 42,15, *p* = 0.0006) but not in SC (F(1,14) = 4.411, *p* = 0.0543).

Analysis of synaptic compartment size revealed further region- and age-specific effects. We observed age effect in vGlut1 size but only in CA3 region (F(1,50) = 4.386, *p* = 0.0413, Figure 1E). Genotype effect was observed in in SC (F(1,50) = 6.450, *p* = 0.0143) and CA1 (F(1,28) = 8.192, *p* = 0.0079, Figure 1E) while age and genotype effect was observed in V1 L5 (F(1,50) = 46.09, *p* < 0.0001), CA1 (F(1,22) = 18.21, *p* = 0.0003) and CA3 (F(1,50) = 4.838, *p* = 0.0325, Figure 1E). Moreover, in V1 L5, vGlut1 puncta size was increased in middle-aged control mice compared to mature adults control mice (*p* = 0.0002). In mature adults, vGlut1 puncta was bigger in Snap25 cKO mice compared to controls in V1 L5 (*p* < 0.0001), CA1 (*p* < 0.0001) and CA3 (*p* = 0.0173). In Snap25 cKO middle-aged mice, vGlut1 puncta was smaller in V1 L5 (*p* = 0.0004) and SC (*p* = 0.0070).

We also observed changes in PSD95 puncta size. Age and genotype effect was only observed in SC (F(1,50) = 5.338, *p* = 0.0250, Figure 1F) and age effect was only observed in CA1 (F(1,50) = 4.548, *p* = 0.0379, Figure 1F) while no genotype effect was observed. In the control brains, PSD95 puncta size was increased in middle-aged mice V1 L5 compared to mature adults (*p* = 0.0456). In middle-aged mice, we observed decrease in PSD95 puncta size in Snap25 cKO mice but only in SC region (*p* = 0.0070).

Together, these findings demonstrate that loss of Snap25-dependent evoked release remodels synaptic architecture in a region- and age-dependent manner, with robust local effects at silencing sites and delayed or selective changes in downstream projection regions. These region- and age-dependent differences suggest that early increases (16 weeks) in synaptic density may reflect compensatory structural remodeling in response to reduced neurotransmission, whereas later-stage (8 months) changes may indicate progressive circuit destabilization or degeneration.

### Synaptic silencing induces region-specific astrocyte reactivity and heterogeneity

To examine how circuit position influences astrocyte responses, we focused on regions representing distinct levels of connectivity to the silenced neuronal populations: the primary somatosensory cortex (S1), which contains directly silenced layer 5 neurons and their silenced local connections; CA3, a major direct projection target of Cre-expressing dentate gyrus cells and CA1, which does not receive direct input from these silenced populations and therefore represents an indirectly affected region. Astrocyte density and astrocyte reactivity were assessed as distinct parameters. GFAP^+^ cell density was used to quantify changes in astrocyte number and/or GFAP expression, whereas GFAP/S100B co-expression was used as a proxy for reactive state, although these markers do not fully capture the spectrum of astrocyte activation.

**Figure 2:**
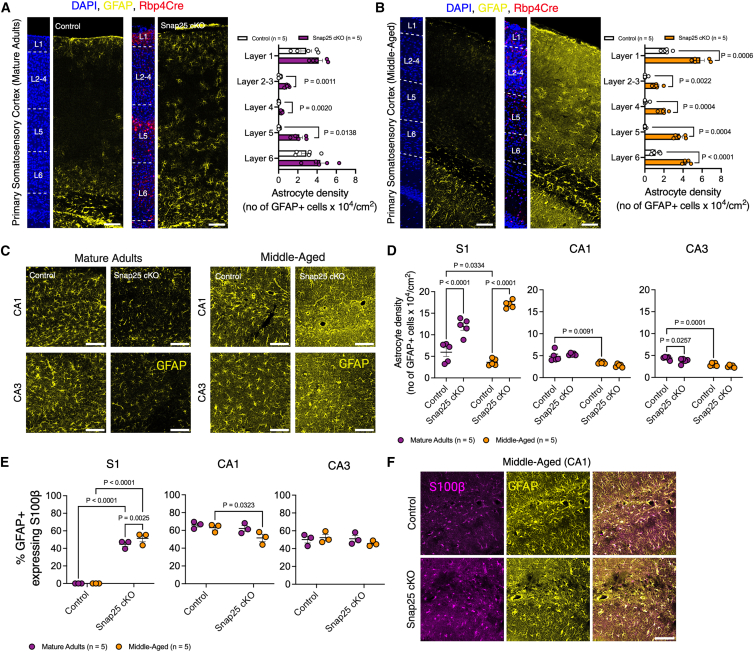
Astrocyte changes in density and marker expression consistent with altered reactive states (A) Representative images of the primary somatosensory cortex (S1) in mature adult mice. DAPI (blue), astrocytes (yellow), and Rbp4^Cre^-expressing layer 5 neurons (red) are shown. Significant changes in astrocyte density were observed across cortical layers. White: control (*n* = 5 animals); purple: Snap25 cKO (*n* = 5 animals). Data were analyzed using mixed-effects ANOVA with Šidák’s multiple comparisons test. (B) Representative images of S1 in middle-aged mice. DAPI (blue), astrocytes (yellow), and Rbp4^Cre^-expressing neurons (red) are shown. Significant changes in astrocyte density were detected across cortical layers. White (control), orange (Snap25 cKO) *n* = 5 for all groups. Data were analyzed using mixed-effects ANOVA with Šidák’s multiple comparisons test. (C) Representative images of astrocytes in the CA1 and CA3 hippocampal regions in mature adult and middle-aged mice. (D) Quantification of astrocyte density in S1, CA1, and CA3. Purple: mature adults (*n* = 5 control; *n* = 5 Snap25 cKO); orange: middle-aged (*n* = 5 control; *n* = 5 Snap25 cKO). Data were analyzed using mixed-effects ANOVA with Fisher’s LSD test. (E) Co-expression of GFAP/S100β in S1, CA1, and CA3. GFAP^+^ density reflects astrocyte number and/or GFAP expression levels, while GFAP/S100B co-expression was used here as an indicator of marker-defined reactivity. Mature adults (purple): *n* = 4–6, and middle-aged (orange): *n* = 4–6. Data were analyzed using mixed-effects ANOVA with Fisher’s LSD test. (F) Representative images of GFAP/S100β co-expression in the CA1 region of middle-aged control and Snap25 cKO mice. GFAP (yellow) and S100β (magenta). All data are presented as mean ± SEM. Statistical significance is indicated in the images. Scale bars (A, B, C, and F): 100 μm.

We first examined age-related changes in control mice. GFAP^+^ astrocyte density was significantly lower in middle-aged controls compared with young/mature adults in S1 (*p* = 0.0334), CA1 (*p* = 0.0091), and CA3 (*p* = 0.0011; Figure 2D), indicating a general age-related decline in astrocyte density across these regions.

We next assessed the effects of Snap25 deletion. In S1, Snap25 loss significantly increased GFAP^+^ astrocyte density at both ages (genotype effect: F(1,8) = 156.4, *p* < 0.0001), with marked alterations in laminar distribution. In young/mature adult mice, GFAP^+^ density was increased in layers 2–5 (L2–3: *p* = 0.0011; L4: *p* = 0.0022; L5: *p* = 0.0138; Figure 2A). In middle-aged mice, GFAP^+^ density was elevated across all cortical layers (L1–L6; all *p* = 0.0006; Figure 2B), indicating a broad redistribution rather than a layer-restricted response.

In the hippocampus, astrocytic responses differed by region. In CA3, a direct projection target of silenced dentate granule cells, GFAP^+^ density was significantly altered by genotype (F(1,8) = 6.078, *p* = 0.0390), with reduced density observed in young/mature adult *Snap25* cKO mice (*p* = 0.0257; Figure 2D). In contrast, GFAP^+^ density in CA1—an indirectly affected downstream region—was not significantly altered by *Snap25* loss (F(1,8) = 0.1107, *p* = 0.7479).

Analysis of GFAP/S100B co-expression revealed further age- and region-dependent effects. In S1, co-expression increased with age (F(1,12) = 146.8, *p* < 0.0001), with higher levels in middle-aged controls compared with mature adults (*p* < 0.0001) and a further increase in middle-aged Snap25 cKO mice (*p* = 0.0005; Figure 2E). In CA1, a significant genotype × age interaction was observed (F(1,16) = 7.717, *p* = 0.0134), with increased co-expression in mature adult Snap25 cKO mice (*p* = 0.0025) and in middle-aged controls relative to mature adult controls (*p* = 0.0210). In CA3, Snap25 deletion significantly increased GFAP/S100B co-expression (F(1,8) = 29.01, *p* = 0.0007), particularly in mature adults (*p* < 0.0001).

These results suggest that astrocyte reactivity may be influenced by circuit position, with pronounced responses in directly silenced or directly innervated regions and comparatively modest changes in indirectly affected areas. The more pronounced changes in CA3 were consistent with its direct receipt of altered dentate input, whereas the relatively modest changes in CA1 may have reflected indirect network-level effects or compensatory adaptations in the absence of direct synaptic silencing. The divergence between regions and ages suggests that astrocyte responses depend on both circuit context and the progression of neurodegeneration, with early changes potentially reflecting adaptive responses to reduced activity and later changes indicating a shift toward more sustained or reactive states.

**Figure 3:**
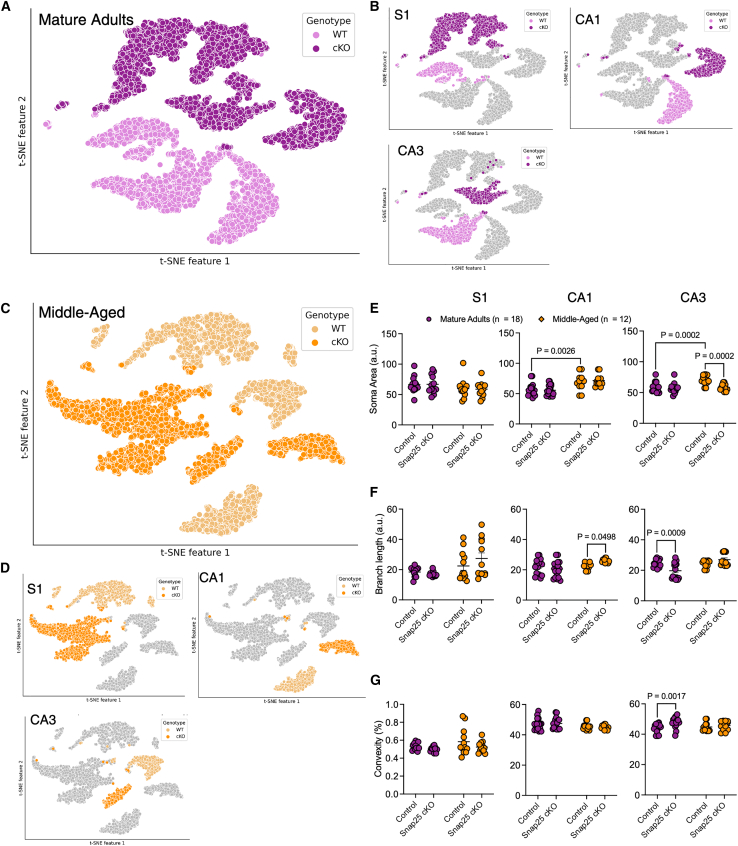
Abolished release of synaptic vesicles affects astrocytes morphology (A–D) t-SNE visualization of astrocyte morphology in control (WT) and Snap25 cKO (cKO) mice. Each point in the t-SNE plots represents a single astrocyte. Purple shades indicate mature adults, and orange shades indicate middle-aged animals. (E–G) Morphological changes in GFAP^+^ astrocytes. Mature adults (purple): *n* = 18 (6 animals per genotype, 3 images per animal; single-cell measurements averaged per image). Middle-aged (orange): *n* = 12 (4 animals per genotype, 3 images per animal; single-cell measurements averaged per image). Data were analyzed using mixed-effects ANOVA with Fisher’s LSD test. All data are presented as mean ± SEM. Statistical significance is indicated in the images.

To evaluate how chronic synaptic silencing affects astrocyte morphology, we measured soma area, branch length, convexity, cell area, Sholl index, and the number of branch points. Data were analyzed using a custom-made algorithm that enables morphological quantification of individual cells, even from lower-magnification images. We used 20× confocal images of astrocytes in S1, CA1, and CA3 regions. In total, we analyzed 10,838 astrocytes at 12–16 weeks (mature adults: control *n* = 4,826 cells; Snap25 cKO *n* = 6,012 cells) and 7,392 astrocytes at 8 months (middle-aged: control *n* = 3,522 cells; Snap25 cKO *n* = 3,870 cells). Astrocyte morphology data were further characterized using t-distributed stochastic neighbor embedding (t-SNE), which incorporated all extracted features to generate maps of morphologically distinct astrocyte populations for comparisons across genotypes, ages, and brain regions (Figures 3A–3D). Detailed statistical data are provided in source data file.

t-SNE analysis of astrocyte morphological features shows that control and Snap25 cKO astrocytes form partially overlapping yet distinct clusters in mature adults, indicating that chronic synaptic silencing alters astrocyte structure (Figure 3A). Region-specific maps reveal that S1 exhibits the greatest range of astrocyte morphologies between genotypes, while CA1 shows partial overlap with more subtle differences, and CA3 displays a broader distribution of cKO astrocytes suggesting greater morphological variability (Figure 3B). In middle-aged animals, t-SNE embeddings again demonstrate clear genotype-dependent clustering, with both control and Snap25 cKO astrocytes forming coherent but distinct groups (Figure 3C). Compared with mature adults, astrocytes from middle-aged mice—particularly in Snap25 cKO animals—showed greater dispersion, indicating increased morphological heterogeneity with age. Snap25 cKO astrocytes formed distinct clusters compared to controls in all regions, with the strongest separation in S1, moderate segregation in CA1, and clear divergence in CA3 (Figure 3B). In S1, the only morphological analysis of GFAP^+^ cells showed changes altered by Snap25 loss. We observed changes in GFAP^+^ cell area, which increased specifically in middle-aged Snap25 cKO mice (*p* = 0.0115; Figures 3E–3G and S2A–S2C). In CA1, we observed age-dependent morphological changes between mature adult and middle-aged controls (soma area: *p* = 0.0026, Figure 3E; cell area: *p* = 0.0027; Figure S2A; number of branch points: *p* = 0.0083; Figure S2C). In middle-aged Snap25 cKO mice, we observed changes in GFAP^+^ cell morphology. In Snap25 cKO, we observed an increase in branch length (*p* = 0.0498, Figure 3F), cell area (*p* = 0.0108, Figure S2A), Sholl index (*p* = 0.0480, Figure S2B), and the number of branch points (*p* < 0.0001, Figure S2C). In CA3, Snap25 loss primarily affected astrocyte morphology in mature adults via alterations in branch length (*p* = 0.0009, Figure 3F), convexity (*p* = 0.0017, Figure 3G), cell area (*p* < 0.0001, Figure S2A), and Sholl index (*p* < 0.0001, Figure S2B). In middle-aged mice, Snap25 loss only impacted GFAP^+^ soma area in CA3 (*p* = 0.0002, Figure 3E), including age-dependent changes (*p* = 0.0002, Figure 3E), and number of branch points (*p* = 0.0092, Figure S2C). Overall, these findings show that chronic suppression of evoked neurotransmission induces distinct astrocyte morphological states that depend on both circuit context and age, with effects of synaptic silencing on astrocytic heterogeneity amplifying over time. The increased morphological heterogeneity observed in middle-aged animals suggests that aging amplifies astrocyte sensitivity to disrupted synaptic activity due to deletion of Snap25, potentially reflecting reduced homeostatic stability or diversification of reactive states.

### Synaptic silencing alters microglial density, morphology, and CD68 expression

We examined how chronic silencing affects microglial cells morphology and dynamics by assessing the organization and density of cells labeled with Iba1 and CD68 in V1, SC, CA1, and CA3 of mature adult and middle-aged mice. These measures reflect changes in microglial density, morphology, and lysosomal marker expression but do not define a specific activation state.

**Figure 4:**
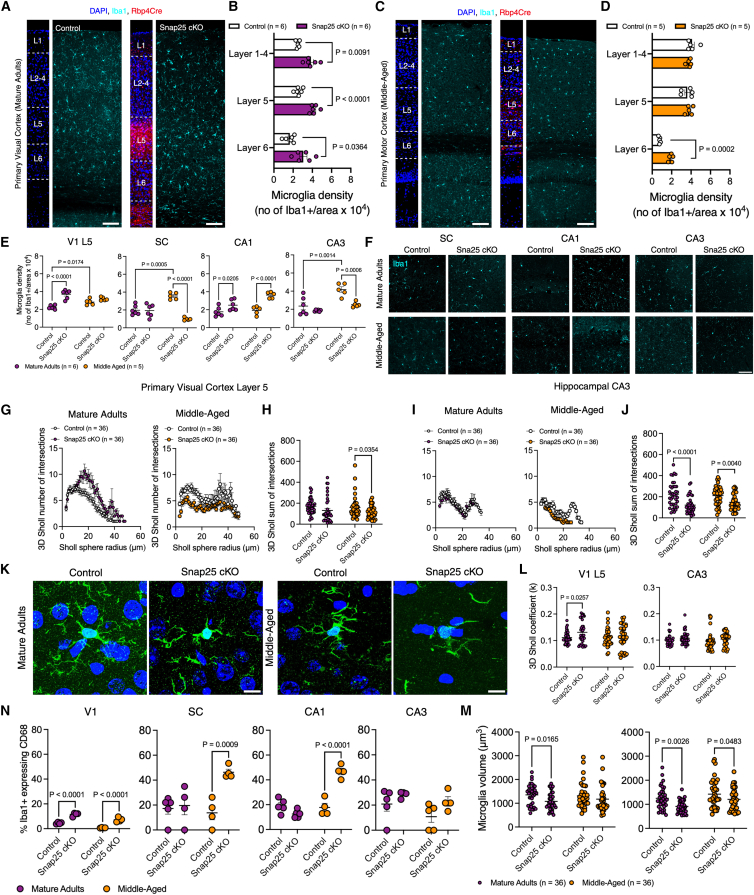
Microglial density, morphology, and CD68 expression are altered following chronic synaptic silencing in Rbp4^Cre^ neuron activity (A–D) Representative images of Iba1^+^ microglia (cyan), DAPI (blue), and Rbp4^Cre^-expressing neurons (red) in primary visual cortex (V1). Microglial density across cortical layers is shown for control (white) and Snap25 cKO mice in mature adults (purple) and middle-aged animals (orange). Data were analyzed using mixed-effects ANOVA with Šidák’s test; *n* = 6 (mature adults, both genotypes) and *n* = 5 (middle-aged, both genotypes). (E) Microglial density in V1, SC, CA1, and CA3. Data were analyzed using mixed-effects ANOVA with Fisher’s LSD test; *n* = 6 (mature adults) and *n* = 5 (middle-aged). (F) Representative images of microglial density in SC, CA1, and CA3. (G–J) 3D Sholl intersections of microglial processes. Data were analyzed using mixed-effects ANOVA with Fisher’s LSD test; *n* = 36 (single cells per age and genotype). (K) Representative images of microglial cells (green) in mature adults and middle-aged control and Snap25 cKO mice brain. (L) Changes in 3D Sholl coefficient between control and Snap25 cKO mice. Data were analyzed using mixed-effects ANOVA with Fisher’s LSD test; *n* = 36. (M) Microglial cell size. Data were analyzed using mixed-effects ANOVA with Fisher’s LSD test; *n* = 36. (N) Iba1/CD68 co-expression in V1, SC, CA1, and CA3. Mature adults (purple): *n* = 4–5 per genotype. Middle-aged (orange): n = 4–5 per genotype. Data were analyzed using mixed-effects ANOVA with Fisher’s LSD test. All data are presented as mean ± SEM. Statistical significance is indicated in the images. Scale bars (A, C, F, and K): 100 μm.

In mature adults (Figures 4A and 4B), Snap25 cKO mice showed increased microglial density across V1 layers (F(1,10) = 44.58, *p* < 0.0001), with the strongest increase in layer 5 (L1–4: *p* = 0.0091; L5: *p* < 0.0001; L6: *p* = 0.0364). In middle-aged Snap25 cKO mice (Figures 4C and 4D), microglial density also increased across V1 layers (F(1,24) = 6.815, *p* = 0.0153), with a notably higher density in L6 (*p* = 0.0002). Across regions, genotype × age interactions were detected in V1, SC, and CA1 but not CA3 (V1: F(1,8) = 15.91, *p* = 0.0040; SC: F(1,8) = 39.91, *p* = 0.0002; CA1: F(1,8) = 7.73, *p* = 0.0239; CA3: F(1,8) = 4.595, *p* = 0.0644). Age-related increases in microglial density were also observed in control mice in V1 (*p* = 0.0174), SC (*p* = 0.0005), and CA3 (*p* = 0.0014). Microglial density was significantly higher in V1 and CA1 of Snap25 cKO mature adults compared to controls (V1: *p* < 0.0001; CA1: *p* = 0.0205; Figure 4E). In middle-aged mice, changes were restricted to subcortical regions: SC and CA3 showed reduced density (SC: *p* < 0.0001; CA3: *p* = 0.0006), while CA1 showed increased density (*p* < 0.0001).

We next assessed microglia-specific 3D morphology. High-resolution 3D microglial morphology analysis was restricted to V1 layer 5 and CA3, as these regions showed the most pronounced genotype-dependent differences in density and represent areas directly affected by synaptic silencing or strong projection input. This targeted approach allowed detailed structural analysis in regions of greatest effect (Figure 4K). Microglial morphology was analyzed at the single-cell level following segmentation of individual Iba1^+^ cells. Cells were sampled in an unbiased manner from each field of view based on Iba1 signal, without manual pre-selection. Given the substantial heterogeneity in microglial morphology, individual cells were retained as independent observations to capture the full range of phenotypic variation. Statistical analysis accounted for the hierarchical structure of the data by grouping cells by animal using mixed-effects models.

This analysis revealed reduced process complexity and smaller cell size in these regions as a result of the loss of Snap25. Chronic silencing led to fewer microglial processes in V1 and CA3 (Figures 4G and 4I), particularly in middle-aged mice. In V1 L5, process number decreased in Snap25 cKO animals (F(1,70) = 8.129, *p* = 0.0057) with a significant reduction in middle-aged mice (*p* = 0.0354; Figure 4H). Similar reductions occurred in CA3 (F(1,70) = 22.45, *p* < 0.0001) in both mature adults (*p* < 0.0001) and middle-aged mice (*p* = 0.0040; Figure 4J). Sholl analysis revealed more complex microglial processes only in V1 L5 of Snap25 cKO mature adults (*p* = 0.0257; Figure 4L). Loss of Snap25 also reduced overall microglial size in V1 L5 (F(1,140) = 6.832, *p* = 0.0099) with a significant reduction in mature adults (*p* = 0.0165; Figure 4M). In CA3, microglia were smaller in Snap25 cKO mice (F(1,140) = 12.81, *p* = 0.0005) in both mature (*p* = 0.0026) and middle-aged groups (*p* = 0.0483). Finally, we assessed the CD68 expression. Snap25 cKO mice showed increased Iba1/CD68 co-expression in cKO mice compared with controls in V1, CA1, and CA3 but not SC (V1: F(1,6) = 84.25, *p* < 0.0001, SC: F(1,7) = 3.403, *p* = 0.1076, CA1: F(1,13) = 11.03, *p* = 0.0055, CA3: F(1,14) = 20.72, *p* = 0.0005, Figure 4N). In mature adults, co-expression increased only in Snap25 cKO V1 (*p* < 0.0001) compared to controls. In middle-aged mice, co-expression increased in Snap25 cKO V1 (*p* < 0.0001), CA1 (*p* = 0.0009), and CA3 (*p* < 0.0001). These results indicate that microglia respond dynamically to sustained disruption of evoked neurotransmission, with region- and age-specific shifts in density, morphology, and lysosomal engagement as indicated by CD68 co-localization. The more pronounced genotype-dependent effects in middle-aged mice suggest an age-related increase in microglial responsiveness to chronic synaptic disruption, potentially reflecting reduced homeostatic stability or cumulative circuit changes.

**Figure 5:**
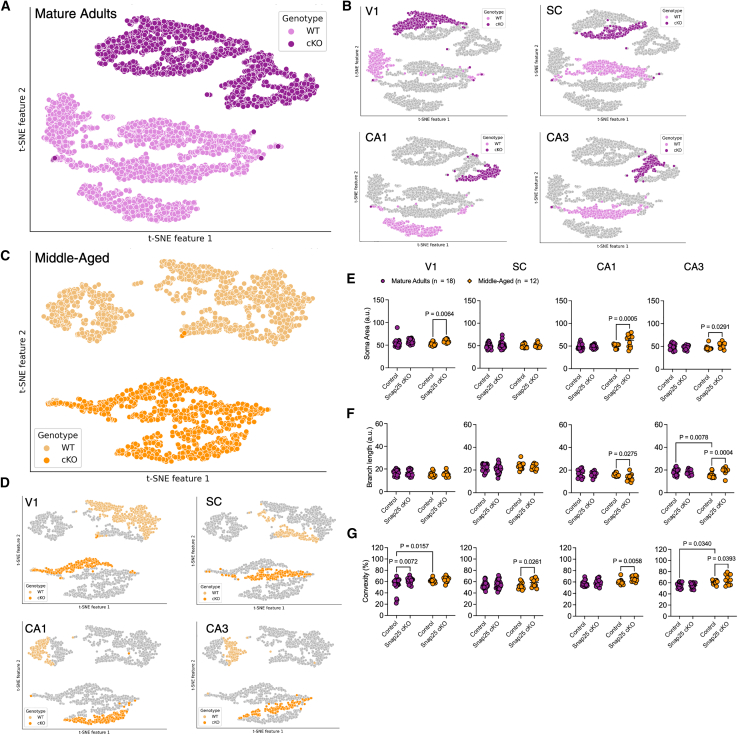
Chronic synaptic silencing affects microglial morphology (A–D) t-SNE visualization of microglial cells morphology in control (WT) and Snap25 cKO (cKO) mice. Each point in the t-SNE plots represents a single microglial cell. Purple shades indicate mature adults, and orange shades indicate middle-aged animals. (E–G) Morphological changes in Iba1^+^ microglia. Mature adults (purple): *n* = 18 (6 animals per genotype, 3 images per animal; single-cell measurements averaged per image). Middle aged (orange): *n* = 12 (4 animals per genotype, 3 images per animal; single-cell measurements averaged per image). Data were analyzed using mixed-effects ANOVA with Fisher’s LSD test. All data are presented as mean ± SEM. Statistical significance is indicated in the images.

Using 20× confocal images, we measured microglial cells’ morphological features in V1, SC, CA1, and CA3. In total, we analyzed 8,616 microglia at 12–16 weeks (mature adults: control *n* = 4,685 cells; Snap25 cKO *n* = 3,931 cells) and 6,137 microglia at 8 months (middle aged: control *n* = 3,017 cells; Snap25 cKO *n* = 3,120 cells). We visualized multivariate morphological relationships using t-SNE (Figures 5A–5D). Detailed statistical data are provided in source data file.

In mature adults, microglia form two largely distinct clusters, with control/WT cells (light purple) and Snap25 cKO cells (dark purple) showing minimal overlap (Figure 5A). This genotype-dependent segregation is preserved in all regions (Figure 5B). In V1, we first assessed age-related changes in control mice. Compared with mature adults, middle-aged control mice exhibited a reduced microglial cell area (*p* = 0.0085; Figure S3A), increased branch complexity as measured by the Sholl index (*p* = 0.0072; Figure S3B), and a reduced number of branch points (*p* = 0.0278; Figure S3C), indicating age-dependent remodeling of microglial morphology. Genotype-dependent effects in V1 were more limited and age specific. In middle-aged Snap25 cKO mice, microglial soma area was significantly greater than that of age-matched controls (*p* = 0.0064; Figure 5E), whereas in mature adult Snap25 cKO mice, microglial convexity was increased relative to controls (*p* = 0.0072; Figure 5G). In SC, genotype effects were detected only in middle-aged mice, where microglial convexity was increased in Snap25 cKO animals compared with controls (*p* = 0.0261; Figure 5G). No significant genotype-dependent differences were observed in mature adults. In CA1, age-related changes were again evident in control mice, with middle-aged controls displaying reduced microglial cell area (*p* = 0.0085; Figure S3A) and fewer branch points (*p* = 0.0025; Figure S3C) compared with mature adults. Genotype-dependent effects emerged selectively in middle-aged Snap25 cKO mice, which showed increased soma area (*p* = 0.0005; Figure 5E), reduced branch length (*p* = 0.0275; Figure 5F), and increased convexity (*p* = 0.0058; Figure 5G) relative to age-matched controls. A similar pattern was observed in CA3, where genotype-dependent changes restricted to middle-aged animals included increased microglial soma area (*p* = 0.0291; Figure 5E), increased branch length (*p* = 0.0004; Figure 5F), and increased convexity (*p* = 0.0393; Figure 5G) in Snap25 cKO mice relative to controls. Age-related changes were also present in CA3, with middle-aged control mice showing reduced branch length (*p* = 0.0078; Figure 5F) and reduced convexity (*p* = 0.0340; Figure 5G) relative to mature adults. Together, these findings indicate that aging is associated with baseline remodeling of microglial morphology across regions, while loss of Snap25-dependent synaptic release selectively amplifies or reshapes these age-related changes. The restriction of genotype effects largely to middle-aged mice suggests that microglia become increasingly sensitive to disrupted synaptic activity with age, potentially reflecting reduced homeostatic resilience or altered reactivity-associated thresholds in the aging brain.

### Loss of Snap25 affects glial cells in the spinal cord

**Figure 6:**
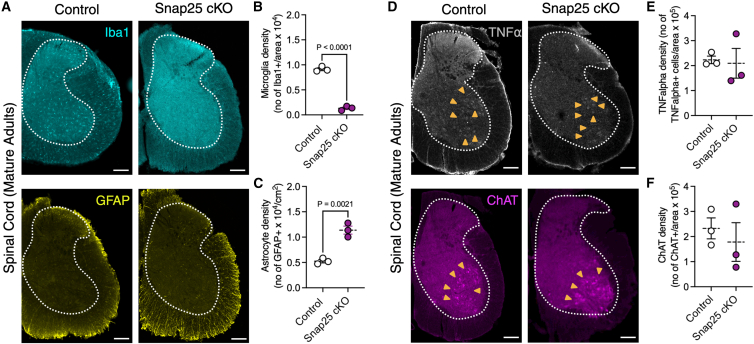
Changes in glial cell density in Snap25 cKO spinal cord (A) Representative images of microglia (cyan) and astrocytes (yellow) in the spinal cord. (B and C) Quantification of Iba1^+^ and GFAP^+^ cell density in control and Snap25 cKO spinal cord (*n* = 3 animals per genotype; unpaired *t* test). (D) Representative images of TNF-α (gray) and ChAT (magenta) immunoreactivity in the ventral horn of the spinal cord. (E and F) TNF-α^+^ and ChAT^+^ cell densities show no significant differences between genotypes (*n* = 3 animals per genotype; unpaired *t* test). All data are presented as mean ± SEM. Statistical significance is indicated in the images. Scale bars (A and D): 200 μm.

A previous study reported neurodegeneration of Rbp4-Cre-expressing neurons in the Snap25 cKO spinal cord (Hoerder-Suabedissen et al., 2019). To further investigate how chronic synaptic silencing influences the spinal cord microenvironment, we examined glial populations (Iba1^+^ microglia and GFAP^+^ astrocytes), neuroinflammatory signaling (TNF-α), and ventral horn motor neurons (ChAT^+^, choline acetyltransferase) in mice aged 13–22 weeks (mature adults; Figures 6A and 6D).

We next examined glial composition and inflammatory markers in the spinal cord, given previous reports of degeneration of Rbp4-Cre-expressing corticospinal neurons in the Snap25 cKO model. In control animals, microglia were uniformly distributed across both white and gray matter. In Snap25 cKO mice, however, overall microglial density was significantly reduced (t = 20.24, df = 4, *p* < 0.0001; Figure 6B). In contrast, GFAP^+^ astrocytes were significantly increased in the spinal cord of Snap25 cKO mice (t = 7.119, df = 4, *p* = 0.0021; Figure 6C), with a preferential enrichment in white matter compared to gray matter, indicating a marked shift in glial composition following loss of Snap25-dependent neurotransmission. To determine whether these changes were accompanied by overt neuroinflammatory signaling or motor neuron pathology, we assessed TNF-α and ChAT immunoreactivity, both of which are commonly associated with inflammatory motor neuron degeneration in conditions such as amyotrophic lateral sclerosis. TNF-α and ChAT immunoreactivity were restricted to the gray matter of the ventral horn. Importantly, TNF-α levels did not differ between genotypes (t = 0.2230, df = 4, *p* = 0.8344; Figure 6E), and ChAT^+^ motor neuron density was similarly unaffected (t = 0.6212, df = 4, *p* = 0.5681; Figure 6F).

Together, these findings indicate that chronic disruption of Snap25-dependent synaptic transmission leads to substantial remodeling of spinal cord glial populations in the absence of detectable TNF-α-mediated neuroinflammation or motor neuron loss. This suggests that glial reorganization in the Snap25 cKO spinal cord occurs independently of overt inflammatory signaling and precedes, or occurs without, measurable degeneration of ChAT^+^ motor neurons. These findings indicate that glial responses to Snap25-dependent synaptic disruption vary across regions of the central nervous system, likely reflecting differences in cellular composition, circuit organization, and regional sensitivity to the loss of upstream synaptic input. Notably, age-by-genotype interactions may be especially pronounced in downstream regions due to progressive degeneration: areas with Rbp4-Cre expression may show more immediate glial responses, whereas projection targets such as the spinal cord may exhibit changes only at later stages.

## Discussion

Our findings support a role for Snap25-dependent vesicular release in shaping neuron-glial interactions. This study demonstrates that chronically silencing regulated neurotransmitter release profoundly alters microglial and astrocytic states across cortical and hippocampal circuits. By abolishing Snap25-dependent release in Rbp4Cre-expressing layer 5 neurons and in the mossy fibers of dentate gyrus granule cells, we were able to compare local and projection-specific changes in downstream targets such as SC and CA3. While the observed regional patterns are consistent with disruption of well-defined projection pathways (e.g., dentate gyrus to CA3 and cortical layer 5 projections to subcortical targets), we cannot exclude contributions from indirect circuit effects or alterations in inputs from other brain regions. Because CA1 does not directly receive inputs from the silenced populations, changes in CA1 reflect indirect effects that are not driven by local synaptic silencing. Importantly, because Snap25 deletion leads to both synaptic silencing and progressive circuit alterations, we cannot definitively distinguish between direct local effects and secondary changes arising from altered network activity or degeneration.

The Rbp4-Cre;Snap25 cKO model has been previously characterized and primarily targets corticofugal layer 5 neurons and dentate gyrus granule cells.^43^^,^^45^ Animals remain viable with largely preserved brain development despite loss of evoked neurotransmission. However, Cre expression is not entirely restricted, and recombination in additional populations, including hypothalamic neurons, cannot be excluded. Consequently, indirect systemic effects may contribute to the observed glial phenotypes. Our recent work using acute chemogenetic manipulation of the same neuronal populations demonstrated that rapid changes in neuronal activity are sufficient to induce bidirectional alterations in synaptic density and glial reactivity,^46^ supporting a direct activity-dependent component to neuron-glia interactions that is independent of long-term degeneration.

Synaptic density changes aligned closely with the topology of neuronal projections. Directly silenced cortical layer 5 exhibited substantial structural reorganization, including increased synaptic density and widespread GFAP upregulation. These effects are consistent with known activity-dependent astrocyte plasticity, in which reduced neuronal firing leads to increased astrocytic coverage, altered synaptic ensheathment, and shifts in metabolic support.^16^^,^^30^ In projection regions such as SC and CA3, synaptic density also increased—specifically in middle-aged SC and in both CA1 and CA3 of mature adults—indicating that presynaptic silencing remodels downstream synaptic architecture. These findings parallel previous reports that synaptic weakening or denervation triggers compensatory structural plasticity and differential astrocyte engagement.^4^^,^^17^ It is important to note that synaptic density and puncta size provide structural measures of synaptic organization but do not fully capture synaptic function or activity levels. Consequently, the absence of a direct correspondence between synaptic density and astrocyte changes does not exclude activity-dependent regulation. Astrocytes are sensitive to multiple aspects of neuronal signaling, including neurotransmitter release dynamics, network activity, and metabolic coupling, which may be altered independently of gross synaptic density.

Astrocytes exhibited robust and region-specific reactivity. GFAP density increased across nearly all cortical layers of S1 at both ages, consistent with direct loss of cortical activity. In contrast, CA1 showed minimal GFAP changes, supporting the interpretation that astrocytes in indirectly affected circuits respond more subtly. CA3 displayed both age- and genotype-dependent effects; notably, Snap25 cKO astrocytes in mature adults showed increased branching and higher cellular complexity, whereas astrocytes in middle-aged mice primarily showed soma enlargement. The differential astrocyte responses observed between CA3 and CA1 further support the idea that circuit connectivity shapes glial remodeling. CA3, as a direct target of dentate granule cells, exhibited more pronounced structural and marker-defined changes, whereas CA1, lacking direct input from the silenced dentate population, showed comparatively subtle alterations. However, these differences may also reflect secondary network effects or compensatory processes rather than purely direct versus indirect mechanisms. These data suggest that astrocytes enter distinct morphological or functional states depending on whether they experience direct loss of activity (S1), loss of presynaptic input and structural remodeling (CA3), or network-level alterations (CA1). The expansion of S100β/GFAP co-expression—particularly in middle-aged S1 and CA3—indicates a shift toward inflammatory or metabolically reprogrammed states, consistent with age-related susceptibility to gliosis.^33^^,^^34^ However, it is important to note that GFAP and S100B expressions reflect only selected aspects of astrocyte reactivity and do not define specific functional states.

t-SNE analyses of astrocyte morphological features further supports this view. Chronic synaptic silencing through loss of Snap25 produced clear genotype-dependent differences that varied across brain regions and ages. In mature adults, control and Snap25 cKO astrocytes occupied partially overlapping but distinct regions of the t-SNE space, consistent with measurable shifts in overall morphological profiles. Region-specific embeddings revealed that these differences were not uniform: S1 showed the clearest separation between genotypes, CA1 exhibited greater overlap, and CA3 displayed a wider distribution of cKO astrocytes, pointing to regionally diverse responses to synaptic inactivity. Aging is known to increase astrocytic heterogeneity and susceptibility to reactive states, which aligns with our observation of a more dispersed cluster distribution. In middle-aged mice, control and Snap25 cKO astrocytes again segregated into distinct clusters, but the overall pattern was more dispersed than in mature adults. Together, these analyses highlight age- and region-dependent shifts in astrocyte structural organization following chronic synaptic silencing, consistent with a transition toward heterogeneous, partially reactive states driven by loss of Snap25.

Microglia were highly sensitive to synaptic silencing, displaying clear genotype-dependent clustering in t-SNE space and region-specific alterations in density, morphology, and CD68 expression. In V1 L5, chronic silencing increased microglial density and reduced process number, consistent with changes in microglial morphology and lysosomal marker expression often associated with reactive phenotypes.^15^ In SC and CA3, both of which receive strong projections from silenced neurons, microglia showed pronounced morphological divergence in both t-SNE embeddings and single-cell features, including increased soma size and higher convexity. These changes likely reflect microglial responses to presynaptic terminal loss, axonal degeneration, and synaptic instability—phenomena well described in developmental pruning and neurodegenerative contexts.^8^^,^^47^ Increased CD68 expression in CA1 and CA3 of middle-aged Snap25 cKO mice further suggests enhanced phagocytic or lysosomal activity in circuits under chronic synaptic stress, consistent with an inflammatory or disease-associated microglial phenotype.

Interestingly, age interacted strongly with genotype across nearly all glial measures. Middle-aged control animals already showed reduced astrocytic density, altered microglial morphology, and shifts in synaptic organization, aligning with reports that aging increases glial heterogeneity and modulates immune surveillance thresholds.^35^ Chronic silencing amplified these age-related trajectories, driving further morphological divergence and heightened inflammatory signatures in both astrocytes and microglia. This suggests that the aging brain becomes increasingly sensitive to disruptions in regulated neurotransmission with glial populations entering more reactive, heterogeneous, and potentially maladaptive states in response to chronic neurotransmission loss.

In the spinal cord, our findings demonstrate that chronic synaptic silencing caused by Snap25 loss leads to marked alterations in glial cell populations. Consistent with previous reports of Rbp4-Cre-mediated axonal degeneration in the Snap25 cKO spinal cord, we observed a substantial reduction in microglial density, despite their uniform distribution across gray and white matter. This decrease contrasts with the significant increase in GFAP^+^ astrocytes, which were particularly enriched in white matter, indicating that astrocytes and microglia respond differently to prolonged loss of presynaptic release of neurotransmitters. In contrast, TNF-α expression and ChAT^+^ motor neuron density remained unchanged, suggesting that inflammatory signaling and motor neuron abundance are not substantially altered at this stage. Together, these results highlight a distinct glial imbalance in the spinal cord microenvironment following long-term synaptic silencing, characterized by reduced microglial presence and increased astrocytic reactivity.

Overall, our findings reveal that the loss of regulated vesicle release alters glial cell morphology and disrupts spatial organization. Importantly, these region-specific glial and structural changes are unlikely to be isolated but instead reflect broader circuit dysfunction. Our previous studies on this model have reported alterations in motor behavior and sensorimotor processing alongside progressive synaptic and axonal changes^43^^,^^44^^,^^45^^,^^48^ suggesting that the glial remodeling observed here may contribute to functional deficits. The combined alterations across cortical, subcortical, and spinal regions further point to coordinated disruption of motor pathways, linking chronic synaptic disruption to system-level consequences. The differential effects across V1/S1, SC/CA3, and CA1 highlight a potential role for circuit topology, with direct synaptic silencing associated with stronger astrocytic changes and projection regions showing distinct microglial remodeling, although these patterns may also reflect indirect network effects or neurodegeneration. This distinct glial imbalance underscores that activity-dependent signaling shapes glial homeostasis across both cortical and spinal circuits, reinforcing the widespread dependence of glial organization on intact presynaptic function.

### Limitations of the study

Snap25 deletion disrupts synaptic transmission, yet it also leads to progressive circuit alterations, including axonal degeneration, interneuron distribution, and neuronal loss at later stages.^43^^,^^44^^,^^45^^,^^46^^,^^48^^,^^49^ Therefore, the glial changes observed in this study cannot be attributed exclusively to loss of synaptic vesicle release. Instead, they likely reflect a combination of reduced neurotransmission, secondary circuit remodeling, and degenerative processes. Further studies will be required to disentangle these contributions.

While this study provides a comprehensive structural and marker-based characterization of glial responses to chronic disruption of Snap25-dependent synaptic transmission, it does not directly assess functional consequences. Future studies integrating functional approaches, such as calcium imaging, metabolic profiling, spatial transcriptomic analysis, synapse-modulatory assays, and inflammatory signaling analyses will be important to determine how these structural changes translate into altered glial physiology and circuit function.

## Resource availability

### Lead contact

Requests for further information and resources should be directed to and will be provided upon reasonable request by lead author, Auguste Vadisiute (auguste.vadisiute@sjc.ox.ac.uk).

### Materials availability

This study did not generate new unique reagents.

### Data and code availability

• Glial cell counter: https://doi.org/10.6084/m9.figshare.26963578; 3D Sholl: https://doi.org/10.6084/m9.figshare.26963587
• MicroCount code is available on request from the lead contact
• Any additional information required is available from the lead contact upon request

## Acknowledgments

A.V.’s graduate studies were funded by the State Study Foundation of Lithuania, Goodger and Schorstein Scholarship from University of Oxford; A.V. is currently funded through a Junior Research Fellowship with St John’s College, Oxford. M.M. is supported through a Rhodes Scholarship, Clarendon Scholarship, and Goodger and Schorstein Scholarship; F.S. was supported from an Anatomical Society Graduate Studentship awarded to A.H.-S. and Z.M., and Goodger and Schorstein Scholarship from University of Oxford; F.M. is a postdoctoral Fellow supported by an MRC Project Grant (G00900901 Z.M.). The work of A.V. was supported by Research Grants from St John’s College Research Centre no 21138077 (A.V. and Z.M.). V.D.’s graduate studies are supported by the Medical Research Council Doctoral Training Programme at Imperial College London [MR/W00710X/1]. The work in S.V.M.’s lab was supported by the UK Dementia Research Institute Pilot Award [DRI-PP20238] and the Academy of Medical Sciences Springboard Award (SBF009\1188). G.G. is supported by an NIH-OxCam graduate studentship. This research was supported in part by the Intramural Research Program of the 10.13039/100000002National Institutes of Health (NIH). The contributions of the NIH authors were made as part of their official duties as NIH federal employees, are in compliance with agency policy requirements, and are considered Works of the United States Government. However, the findings and conclusions presented in this paper are those of the authors and do not necessarily reflect the views of the NIH or the U.S. Department of Health and Human Services.

## Author contributions

Conceptualization, A.V., F.S., A.H.-S., and Z.M.; experimental design, A.V.; transgenic line development and breeding, A.V., F.S., M.M., and A.H.-S.; tissue collection and preparation, A.V., F.S., S.L, G.G., and M.M.; immunohistochemistry and imaging, A.V., F.S., S.L., G.G., and M.M.; data analysis, A.V., S.L., F.M., V.D., and A.U.; custom-made algorithms, A.V., F.M, V.D., A.U., and S.V.M.; provided funding, A.V., F.S., S.V.M., and Z.M.; wrote the first draft of the paper, A.V. and S.L. All authors discussed the results and commented or edited the paper.

## Declaration of interests

The authors declare no competing interests.

## STAR★Methods

### Key resources table

**Table:** 

REAGENT or RESOURCE	SOURCE	IDENTIFIER
**Antibodies**		

Rabbit Anti-Iab1	FUJIFILM Wako	#019-19741
Chicken Anti-GFAP	Abcam	#ab4674
Mouse Anti-TNFa	Abcam	#ab1793
Mouse Anti-S100b	Sigma-Aldrich	#S2532
Mouse Anti-CD68	Abcam	#ab955
Mouse Anti-vGlut1	Synaptic systems	#135011
Rabbit Anti-PSD95	Abcam	#18258
Rabbit Anti-ChAT	Abcam	#ab178850
Alexa Fluor®488 Goat Anti-Mouse	Abcam	#ab150113
Alexa Fluor®488 Goat Anti-Rabbit	Abcam	#ab150077
Alexa Fluor®633 Goat Anti-Mouse	Thermo Fisher Scientific	#A-21050
Alexa Fluor®647 Goat Anti-Mouse	Abcam	#ab150115
Alexa Fluor®647 Goat Anti-Chicken	Abcam	#ab150171

**Chemicals, peptides, and recombinant proteins**		

4% formaldehyde	Sigma Aldrich	F8775
sodium azide	Sigma Aldrich	26628-22-8
normal goat serum	Sigma Aldrich	G9023
Mowiol	Sigma Aldrich	81381
Prolong Gold	ThermoFisher	P36930

**Experimental models: Organisms/strains**		

B6-Snap25tm3mcw mice (Snap25 fl/fl and Snap25fl/+)	Michael C. Wilson	University of New Mexico
The Rbp4-Cre line (Tg(Rbp4-cre)KL100Gsat/Mmucd)	Jackson Laboratories	https://www.informatics.jax.org/strain/MGI:4367068
Ai14 reporter line (B6;129S6-Gt(ROSA)26Sorˆtm14(CAG-tdTomato)Hze/J)	Jackson Laboratories	https://www.jax.org/strain/007908

**Software and algorithms**		

ImageJ	NIH	Version: 2.16.0/1.54p
JACoP plugin		https://imagej.net/plugins/jacop
MATLAB	MathWorks	https://www.mathworks.com/products/matlab.html
Glial cell counter	https://doi.org/10.6084/m9.figshare.26963578	
3D Sholl analysis	https://doi.org/10.6084/m9.figshare.26963587	
GraphPad Prism v10.2	GraphPad	https://www.graphpad.com

**Other**		

vibratome	Leica VT1000S	
laser-scanning confocal microscope	Zeiss LSM710	
Slide scanner	Evident VS200	

### Experimental model and study participant details

#### Mouse breeding and maintenance

All experimental procedures were conducted in accordance with the UK Animals (Scientific Procedures) Act, 1986 (ASPA), and the University of Oxford’s Policy on the Use of Animals in Scientific Research (PP0546018, P828B64BC). Ethical approval was granted by the University of Oxford Animal Welfare and Ethical Review Board. Animals were housed in individually ventilated cages under a 12-h light/dark cycle, with food and water provided *ad libitum*.

B6-Snap25tm3mcw mice (Snap25 fl/fl and Snap25fl/+) were obtained from the University of New Mexico (Michael C. Wilson) and maintained on a C57BL/6 background. The Rbp4-Cre line (Tg(Rbp4-cre)KL100Gsat/Mmucd) was obtained from Jackson Laboratories, and the Ai14 reporter line (B6;129S6-Gt(ROSA)26Sorˆtm14(CAG-tdTomato)Hze/J) was also obtained from Jackson Laboratories. Experimental and control cohorts were generated by mating female breeders (Cre/+;Snap25fl/+;Ai14/+) with male breeders (Snap25 fl/fl;Ai14/Ai14). This produced offspring with the following genotypes: Cre/+;Ai14;Snap25 fl/fl (Snap25 cKO) and +/+;Ai14;Snap25fl/+ or +/+;Ai14;Snap25 fl/fl (control). The control group lacks Cre expression, and the presence of *LoxP* sites alone does not affect Snap25 gene function (Hoerder-Suabedissen et al., 2019). Sample sizes varied according to animal availability (Table S1).

### Method details

#### Perfusion

Adult mice were anesthetized with 0.6 mL/kg pentobarbital administered via intraperitoneal injection. Animals were transcardially perfused with 0.01 M phosphate-buffered saline (PBS, pH 7.4) followed by 4% paraformaldehyde (PFA) in PBS. Brains were removed and postfixed in 4% PFA (F8775, Sigma Aldrich) overnight at 4 °C, then transferred to 1× PBS with 0.05% sodium azide (26628-22-8; Sigma Aldrich) and stored at 4 °C until sectioning.

#### Brain tissue sectioning

PFA-fixed brains were embedded in 4.5% agarose and cut into 50 μm coronal sections on a vibratome (Leica VT1000S). Sections were transferred to 24-well plates and stored in 0.1 M PBS with 0.05% sodium azide at 4 °C.

#### Immunohistochemistry

All sections, except those used for tumor necrosis factor-alpha (TNF-α) staining, underwent a standard blocking procedure. Sections were incubated for 2 h at room temperature in a blocking solution containing 0.3% Triton X-100 and 5% normal goat serum (NGS) (Sigma-Aldrich). Protocols for spinal cord staining involved blocking in 0.3% Triton X-100 and 10% NGS, followed by an additional aldehyde-quenching wash in 0.1M glycine for 15 min. Primary antibodies (Table S2) diluted in blocking solution were applied overnight at 4 °C, wherein solutions for spinal sections then used 5% NGS. After three PBS washes, sections were incubated for 2 h at room temperature with secondary antibodies (Table S2) diluted in 0.3% Triton X-100 and 5% NGS. All sections were mounted in Mowiol (Sigma-Aldrich, 81381), save spinal sections which were mounted in Prolong Gold (ThermoFisher, P36930).

#### Confocal microscopy

Immunolabeled brain sections were imaged with a laser-scanning confocal microscope (Zeiss LSM710). Spinal sections were imaged using an Evident VS200 slide scanner at 20x resolution (air objective, 0.325 μm pixel size). To image for Iba1^+^ and GFAP^+^ cell density, microglial phagocytic activity and inflammatory marker presence, the 20x/0.6 air objective and 1x optical zoom at 0.42 μm pixel size, and frame size 1024 x 1024 was used. Areas imaged were the somatosensory and visual cortex layer 5, superior colliculus, and the hippocampus CA1 and CA3 regions. A 20x/0.6 air objective with 1x optical zoom, 0.42 μm pixel size, and 1024 x 1024 frame size was used to examine individual microglial cell morphological features and vGlut1/PSD95 synaptic densities.

### Quantification and statistical analysis

#### Synaptic density

vGlut1 and PSD95 particle number and volume were analyzed in ImageJ using the 3D ROI Manager. Background subtraction, 3D Gaussian smoothing, and manual thresholding were applied before 3D reconstruction. Data were normalized to image volume and number of planes. PSD95–vGlut1 co-localization was quantified using JACoP (https://imagej.net/plugins/jacop).

#### Cell counter

Microglial and astrocyte densities were quantified using an automated Python-based cell counter (https://doi.org/10.6084/m9.figshare.26963578) as previously described by A. Vadisiute et al. (2024). GFAP^+^ astrocytes and Iba1^+^ microglia were quantified using a custom image analysis pipeline implemented in Python using the *scikit-image* library (https://scikit-image.org). Images were first preprocessed using Gaussian filtering (skimage.filters) to reduce noise and improve signal detection.

For GFAP^+^ and Iba1^+^ cells, signal intensity thresholding was applied to generate binary masks, followed by connected-component labeling (skimage.morphology) to identify individual signal-positive regions. For Iba1^+^ microglia, segmentation was further refined by incorporating overlap with DAPI-labeled nuclei to improve cell identification specificity.

Individual cells were approximated by separating connected components based on size and morphology, with small objects excluded as noise. Each connected component was counted as a single cell for density measurements within each field of view.

#### 3D analysis

Volume: Iba1-labeled microglia were segmented using 26-neighbor connectivity. Connected components smaller than 300 voxels were removed as noise. Voxel dimensions were taken from confocal imaging metadata (Zeiss LSM710; z = 0.55 μm; x = y = 0.0658941 μm). The Iba1 channel was Gaussian-smoothed (σ = 2) and thresholded.

3D Sholl: Analysis included 3D Gaussian smoothing, thresholding, removal of small components, generation of spherical masks, and counting intersections between masks and cell segmentations at increasing radii. Intersections were mapped to N/S, and linear regression yielded the 3D Sholl coefficient k (https://doi.org/10.6084/m9.figshare.26963587).

#### Microcount methodology

Morphological features of microglia and astrocytes were quantified using a custom-made image analysis algorithm that supports high-throughput single-cell extraction from lower-magnification 2D confocal images. This analytical approach offers a substantial advantage over conventional high-magnification 3D reconstructions: whereas 3D imaging typically yields one analyzable cell per image, the 2D pipeline enables the segmentation and quantification of many glial cells within a single image. Consequently, it provides improved statistical power, greater sampling depth across brain regions, and significantly reduced imaging and processing time while preserving cell-level morphological resolution.

The algorithm used in this study is fully operational but is part of an ongoing project that has not yet been formally released or peer-reviewed. Because the code is still under active development and subject to licensing and documentation considerations, it cannot be publicly shared in full at this stage. Once the associated software manuscript is completed and the codebase is formally deposited in an appropriate open-access repository, the complete version will be made available.

In the meantime, all methodological details relevant for reproducibility—including image preprocessing steps, segmentation parameters, and extracted morphological features—are described in the Methods. Example output files and limited script excerpts can be provided upon reasonable request, subject to institutional guidelines and software release policies.

Morphological analyses of astrocytes and microglial cells in 2D images of control and Snap25 cKO mice at 12–16 weeks and 8 months was performed using Microcount, a MATLAB application for large-scale glial cell morphological analysis (ref). Cells were first segmented based on their soma and branch signal intensity using Iba1 for microglia and GFAP for astrocytes, creating binary cell masks. Following the segmentation of individual cells, several morphological parameters were computed on both a single-cell and an image-wise basis. Morphological parameters included: percentage coverage, cellular density, convexity, soma size, branchpoint count, branch length and the Sholl index.

Percentage coverage was computed as the proportion of the image covered by the binary cell mask. Cell density was calculated as the ratio between the number of identified somas and the image area. Convexity was calculated as the ratio between the area of a cell mask and the area of its minimum bounding polygon, often referred to as the convex hull. Soma size was calculated as the area of each soma within the soma mask. Branchpoint count and branch length were computed using the skeleton of the cell mask. Branch points are defined points within the skeleton of each cell where two or more processes meet.

The Sholl index for each cell was calculated by determining the number of intersections of microglial branches with circles of increasing radius originating from the center of mass of each cell. The intersection counts where then used to compute the Sholl Coefficient (k) using the semi-Log linear regression method in accordance with the following equation:

$$\log\;10\left(\frac{N}{S}\right)=-k\times r+m$$

Where N is the number of crossings at a given radius (r) and S is the area of a circle of radius r.

Finally, co-localization with additional markers, CD68 for microglia and S100B for astrocytes, was determined by thresholding the co-marker signal intensity to generate a binary co-marker mask. Two measures of co-localization were calculated: (1) percentage co-localization by area and (2) the proportion of cells co-localizing with the co-marker by at least 5% for S100b and 1% for CD68.

#### tSNE

To visualize complex multidimensional datasets, dimensionality reduction techniques including principal component analysis (PCA) and t-distributed stochastic neighbor embedding (t-SNE) were used. t-SNE is a non-linear dimensionality reduction method that captures local structure by modeling pairwise similarities using a Student’s *t* distribution in the low-dimensional embedding space (van der Maaten & Hinton, 2008). The balance between local and global data structure is controlled by the perplexity parameter, which reflects the effective number of nearest neighbors considered for each data point.

t-SNE analyses were performed using the Python library *scikit-learn*, with parameters set according to the library’s default recommendations (Pedregosa et al., 2011). t-SNE was used exclusively for exploratory visualization and not for statistical inference.

### Quantification and statistical analysis

All statistical analyses were performed using Prism (GraphPad Prism v10.2). Data were first assessed for normality using the Shapiro–Wilk test. All datasets satisfied criteria for normal distribution and exhibited homogenous variance across groups; thus parametric tests were applied throughout. Sample sizes were determined at the animal level, and imaging datasets consisted of multiple fields or single-cell technical replicates averaged per animal or per image, as specified for each figure. Both male and female mice were used; detailed information for each mouse is provided in Table S1. For synaptic analyses, mature adult groups consisted of *n* = 5 control and *n* = 5 Snap25 cKO mice, and middle-aged groups consisted of *n* = 4 control and *n* = 4 Snap25 cKO mice, and spinal cord analysis consisted of *n* = 3 control and *n* = 3 Snap25 cKO mature adult. Astrocyte and microglial density, GFAP/S100β and Iba1/CD68 co-expression, and t-SNE–derived morphology metrics were quantified from n = 5–6 animals per genotype for mature adults and n = 4–5 animals per genotype for middle-aged mice. For single-cell morphological analyses, data were averaged per image (three images per animal), yielding *n* = 18 samples per group in mature adults and *n* = 12 in middle-aged animals. For 3D microglial Sholl and cell-size analyses, *n* = 36 individual cells per age and genotype were examined. Comparisons across genotype, age, and brain region were conducted using mixed-effects ANOVA to accommodate missing values and hierarchical data structure. When assessing layer-specific or region-specific effects, Šidák’s multiple comparisons test was used; in all other cases, Fisher’s LSD test was applied for post-hoc comparisons. All data are presented as mean ± SEM, and statistical significance was indicated in panels. All figures created using BioRender.com.
