# Supplementary material for: Deletion of Snap25 disrupts glial remodeling in aging mouse brain
Source: iScience. 2026 Jun 23;29(7):116478. doi: 10.1016/j.isci.2026.116478 (PMC13316228; doi:10.1016/j.isci.2026.116478)
Supplement: Document S1. Figures S1–S3 and Tables S1 and S2 [file mmc1.pdf]

**iScience, Volume *29***

## Supplemental information

# Deletion of Snap25 disrupts glial remodeling in aging mouse brain

**Auguste Vadisiute, Florina Szabo, Sofia Luchanskaya, Vanessa Drevenakova, Fernando Messore, Albert Ugwudike, Gretchen Greene, Marissa Mueller, Sophie V. Morse, Anna Hoerder-Suabedissen, and Zoltán Molnár**

**Supplementary information**
**Supplementary tables**
**Table 1: Number of animals**

| Animal ID | Age | Genotype | Sex |
|---|---|---|---|
| SBRT145.2d | 16 weeks | Control | Female |
| SBRT145.2e | 16 weeks | Control | Female |
| SBRT145.2f | 16 weeks | Control | Female |
| SBRT166.3b | 12 weeks | Control | Female |
| SBRT169.1a | 12 weeks | Control | Male |
| SBRT177.1d | 12 weeks | Control | Female |
| SBRT174.2d | 12 weeks | Control | Female |
| SBRT187.5e | 12 weeks | Control | Female |
| SBRT176.2d | 12 weeks | Control | Female |
| SBRT143.2b | 16 weeks | Snap25 cKO | Female |
| SBRT143.2e | 16 weeks | Snap25 cKO | Female |
| SBRT145.2c | 16 weeks | Snap25 cKO | Female |
| SBRT168.2e | 12 weeks | Snap25 cKO | Female |
| SBRT169.1d | 12 weeks | Snap25 cKO | Female |
| SBRT177.1e | 12 weeks | Snap25 cKO | Female |
| SBRT169.1e | 12 weeks | Snap25 cKO | Female |
| SBRT176.2f | 12 weeks | Snap25 cKO | Female |
| SBRT177.1b | 12 weeks | Snap25 cKO | Female |
| SBRT140.4b | 8 months | Control | Male |
| SBRT144.2c | 8 months | Control | Male |
| SBRT144.4d | 8 months | Control | Male |
| SBRT190.1a | 8 months | Control | Male |
| SBRT190.1b | 8 months | Control | Female |
| SBRT140.4a | 8 months | Snap25 cKO | Male |
| SBRT143.4b | 8 months | Snap25 cKO | Female |
| SBRT144.4b | 8 months | Snap25 cKO | Male |
| SBRT188.2a | 8 months | Snap25 cKO | Male |
| SBRT188.3a | 8 months | Snap25 cKO | Male |
| SBRT202.2a | 22 weeks | Snap25 cKO | Male |
| SBRT205.1a | 21 weeks | Snap25 cKO | Male |
| SBRT205.1g | 17 weeks | Snap25 cKO | Female |
| SBRT205.1h | 18 weeks | Control | Female |
| SBRT205.2h | 13 weeks | Control | Female |
| SBRT206.1h | 21 weeks | Control | Female |
| SBRT206.1g | 21 weeks | Snap25 cKO | Female |
| SBRT205.1c | 21 weeks | Snap25 cKO | Male |
| SBRT214.2e | 20 weeks | Control | Female |
| SBRT205.1b | 21 weeks | Control | Male |

**Table 2: Primary and Secondary Antibodies used for immunohistochemical experiments.** Optimal primary antibody concentrations and incubation conditions were determined using spare brain sections. All antibodies were selected based on extensive literature citations and reported specificity.

| Primary Antibodies | | | | |
|---|---|---|---|---|
| **Target** | **Host species** | **Dilution** | **Manufacturer** | **Catalogue Number** |
| Anti-Iba1 | Rabbit | 1:500 | FUJIFILM Wako | #019-19741 |
| Anti-GFAP | Chicken | 1:500 | Abcam | #ab4674 |
| Anti-TNFa | Mouse | 1:250 | Abcam | #ab1793 |
| Anti-S100b | Mouse | 1:250 | Sigma-Aldrich | #S2532 |
| Anti-CD68 | Mouse | 1:500 | Abcam | #ab955 |
| Anti-vGlut1 | Mouse | 1:500 | Synaptic systems | #135011 |
| Anti-PSD95 | Rabbit | 1:500 | Abcam | #18258 |
| Anti-ChAT | Rabbit | 1:500 | Abcam | #ab178850 |
| **Secondary Antibodies** | | | | |
| **Fluorophore** | **Species** | **Concentration** | **Manufacturer** | **Catalogue Number** |
| Alexa Fluor®488 | Goat Anti-Mouse | 1:500 | Abcam | #ab150113 |
| Alexa Fluor®488 | Goat Anti-Rabbit | 1:500 | Abcam | #ab150077 |
| Alexa Fluor®633 | Goat Anti-Mouse | 1:500 | Thermo Fisher Scientific | #A-21050 |
| Alexa Fluor®647 | Goat Anti-Mouse | 1:500 | Abcam | #ab150115 |
| Alexa Fluor®647 | Goat Anti-Chicken | 1:500 | Abcam | #ab150171 |

## Supplementary figures

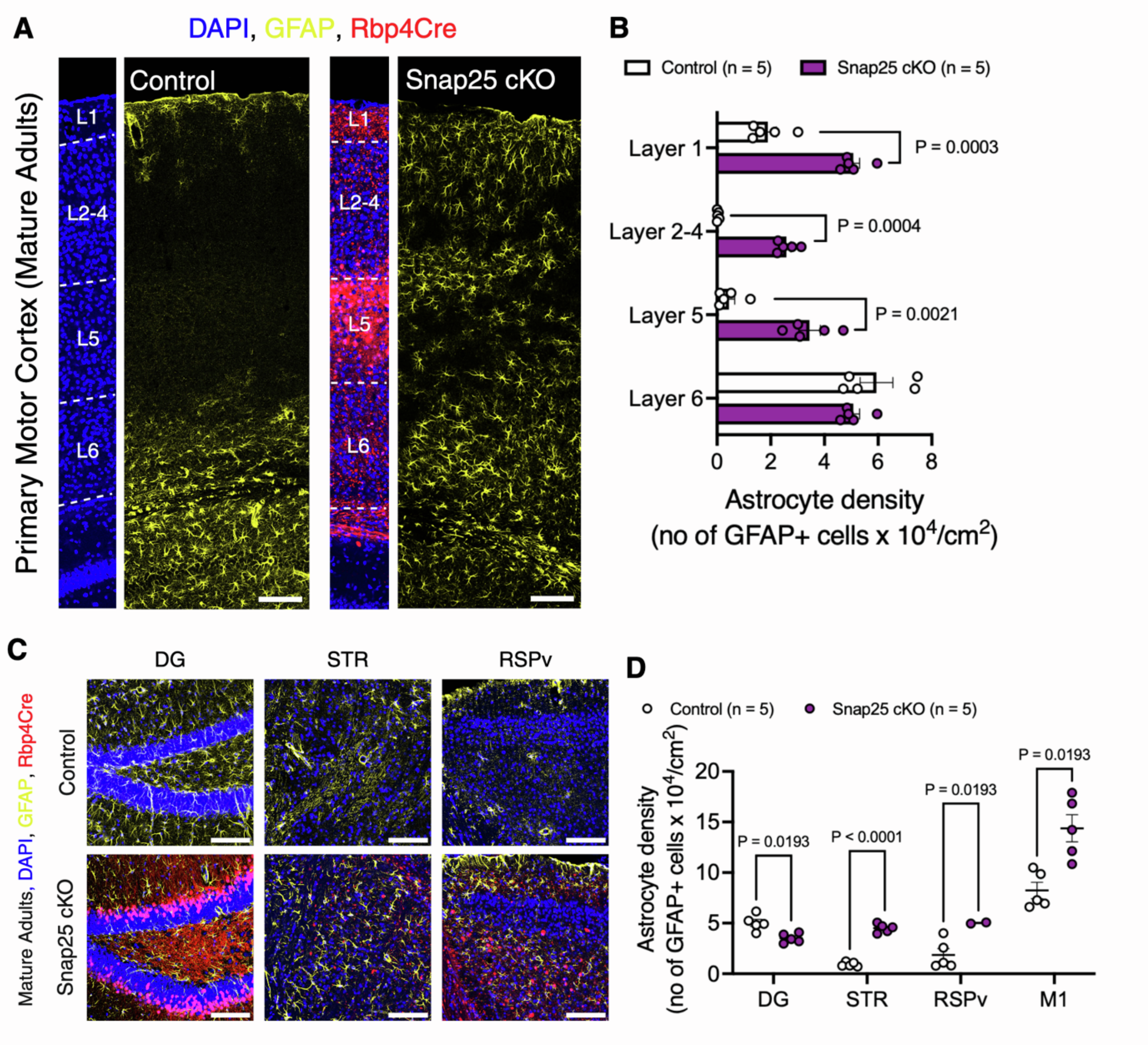


**Supp. Figure 1: Astrocytes respond to chronic synaptic silencing by increasing reactivity and inflammation in cortical and subcortical regions**
**A,C.** Representative images of astrocytes in M1, DG, STR and RSPv in mature adults, DAPI (blue), astrocytes (yellow), $Rbp4^{Cre}$ L5 neurons (red). **B.** Astrocytes density across cortex and different brain regions. Significant changes in astrocyte density were detected across cortical layers. White: control (n=5 animals); purple: Snap25 cKO (n=5 animals). Data were analysed using mixed-effects ANOVA via Šidák's test. **D.** Astrocytes density across cortex and different brain regions. Significant changes in astrocyte density were detected across cortical layers. White: control (n=5 animals); purple: Snap25 cKO (n=5 animals, except RSPv n = 2). Data were analysed using mixed-effects ANOVA with Fisher's LSD test. All data are presented as mean ± SEM. Statistical significance is indicated in panels. Scale bars (A, B, C, F): 100 µm.

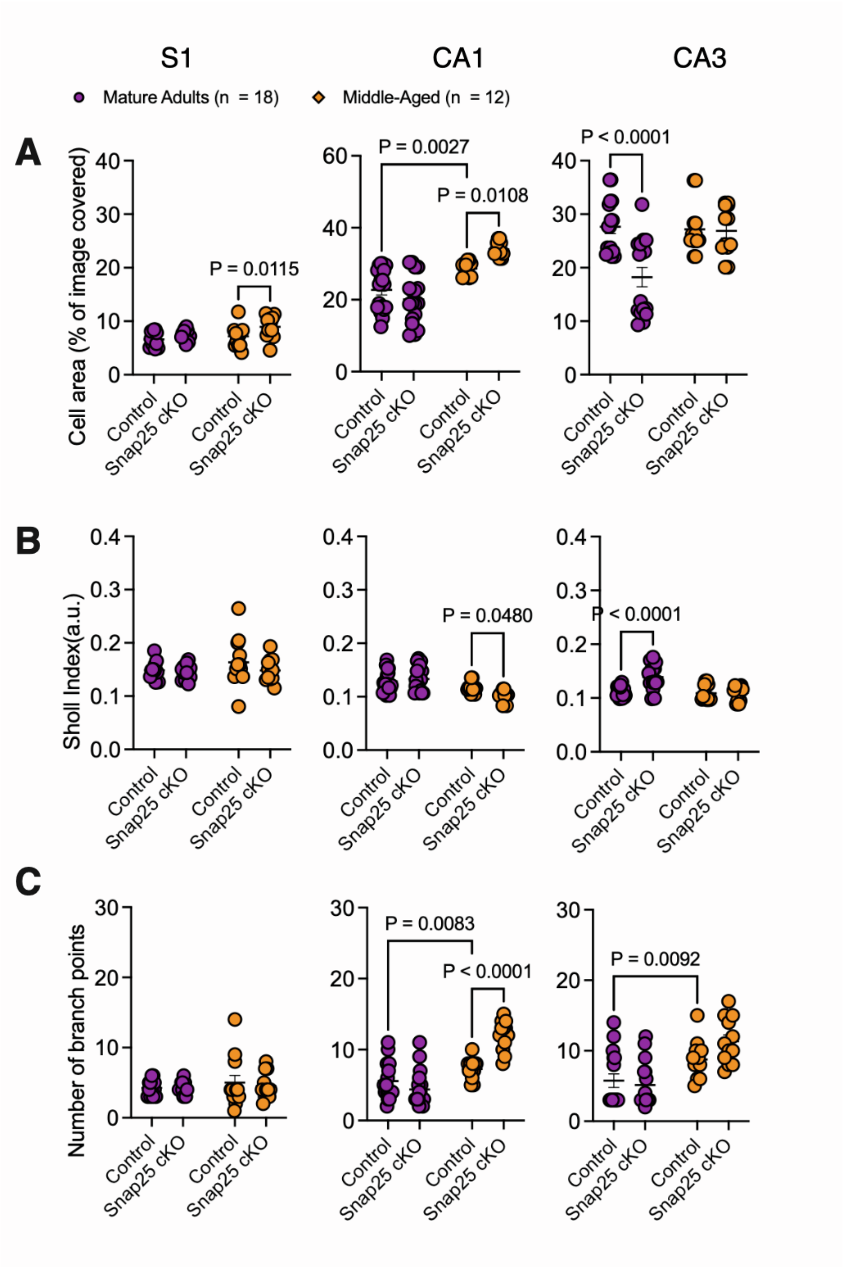


**Suppl. Figure 2: Changes in astrocyte morphology in mature and aging brains**

**A-C.** Morphological changes in GFAP⁺ astrocytes. Mature adults (purple): n = 18 (6 animals per genotype, 3 images per animal; single-cell measurements averaged per image). Middle-aged (orange): n = 12 (4 animals per genotype, 3 images per animal; single-cell measurements averaged per image). Data were analysed using mixed-effects ANOVA with Fisher's LSD test. All data are presented as mean ± SEM. Statistical significance is indicated in panels.

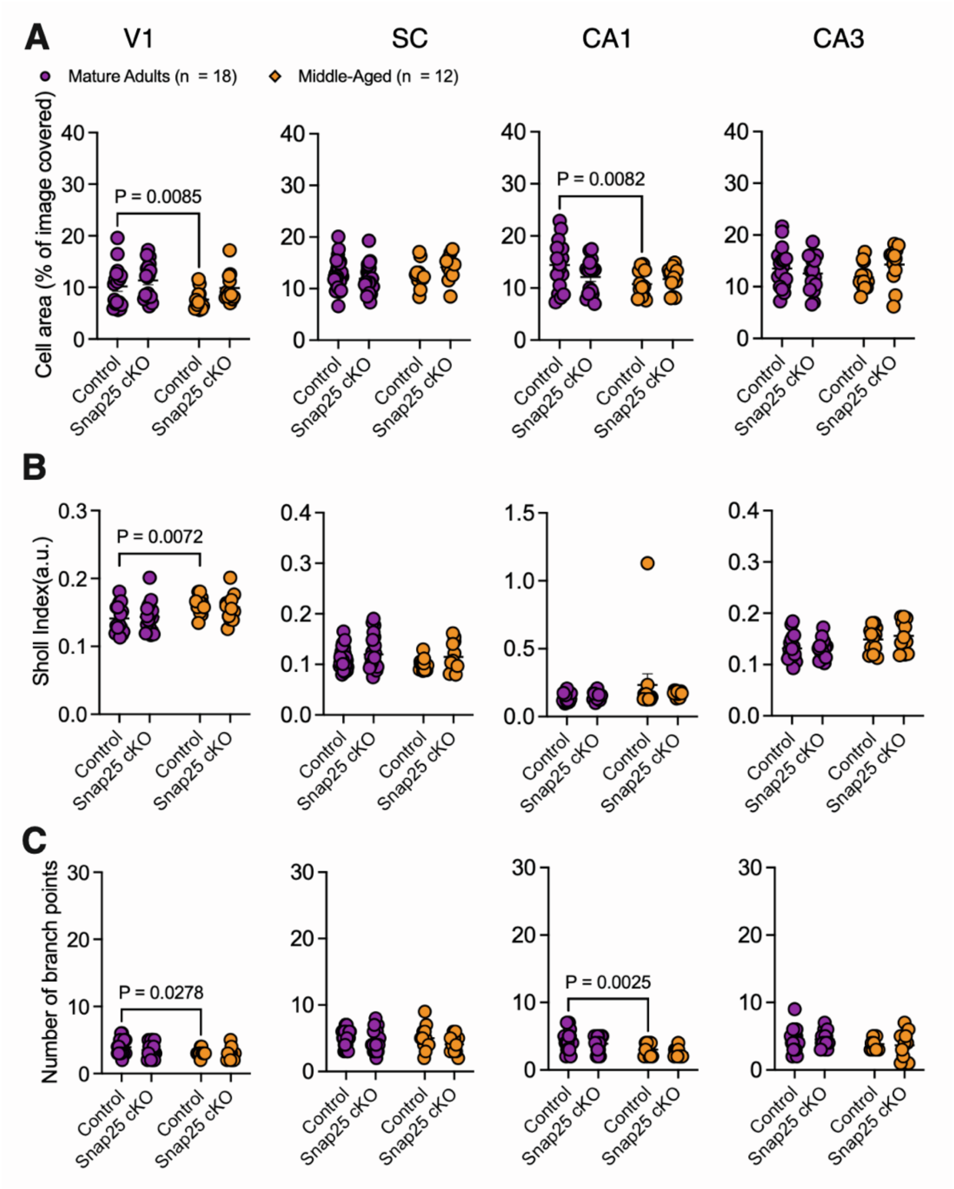


**Suppl. Figure 3: Changes in microglial cells morphology in mature and aging brains**
**A-C.** Morphological changes in Iba1$^+$ microglia. Mature adults (purple): n = 18 (6 animals per genotype, 3 images per animal; single-cell measurements averaged per image). Middle-aged (orange): n = 12 (4 animals per genotype, 3 images per animal; single-cell measurements averaged per image). Data were analysed using mixed-effects ANOVA with Fisher's LSD test. All data are presented as mean ± SEM. Statistical significance is indicated in panels.
